# Perinatal Risks of Neonatal and Infant Mortalities in a Sub-provincial Region of China: A Livebirth Population-based Cohort Study

**DOI:** 10.1186/s12884-022-04653-8

**Published:** 2022-04-19

**Authors:** Yaling Xu, Xiaojing Guo, Zhaojun Pan, Guofang Zheng, Xiaoqiong Li, Tingting Qi, Xiaoqin Zhu, Hui Wang, Weijie Ding, Zhaofang Tian, Haijun Wang, Hongni Yue, Bo Sun, on behalf of the Huai’an Perinatal-Neonatal Collaborative Study Group, Zhaojun Pan, Guofang Zheng, Sufang Ding, Xiaoqiong Li, Tingting Qi, Xiaoqin Zhu, Hui Wang, Weijie Ding, Hongni Yue, Zhaofang Tian, Muling Zhang, Haijun Wang, Yaodong Yin, Honghua Guan, Juan Yang, Yongjian Wu, Tao Xu, Chunhong Tang, Maotian Dong, Chunhua Zhang, Chunqin Dong, Sumei Zhou, Yani Lei, Shouzhong Li, Keyan Zhu, Xia Zhao, Bi Xue, Zhaoxia Wang, Shucheng Wang, Hong Liu, Zhou Xu, Chuntao Yuan, Xihui Cao, Jianya Zhang, Bu Xu, Wenlong Lin, Cui Gao, Yongbo Heng, Lei Wang, Moqing Wang

**Affiliations:** 1 The National Commission of Health Laboratory of Neonatal Diseases; National Children’s Medical Center, Children’s Hospital of Fudan University, Shanghai, China; 2 Department of Neonatology, Huai’an Women and Children’s Hospital, Huai’an, Jiangsu China; 3 Department of Obstetrics, Huai’an Women and Children’s Hospital, Huai’an, Jiangsu China; 4 Unit of Population Health Information, Huai’an Women and Children’s Hospital, Huai’an, Jiangsu China; 5 Department of Neonatology, The Affiliated Huai’an First People’s Hospital of Nanjing Medical University, Huai’an, Jiangsu China; 6 Department of Neonatology, Lianshui County Hospital, Huai’an, Jiangsu China

**Keywords:** Livebirth, Neonate mortality rate, Infant mortality rate, Neonatal hospitalization, Risk

## Abstract

**Background:**

Current vital statistics of birth population and neonatal outcome in China lacked information and definition of deaths at delivery and during hospitalization, especially for extreme preterm (EPT) birth. This study aims to delineate the prevalence of neonatal hospitalization, neonatal and infant mortality rates (NMR, IMR) and associated perinatal risks based on all livebirths in Huai’an, an evolving sub-provincial region in eastern China.

**Methods:**

This retrospective cohort study established a comprehensive database linking information of whole regional livebirths and neonatal hospitalization in 2015, including deaths at delivery and EPT livebirths. The primary outcomes were NMR and IMR stratified by gestational age (GA) and birthweight (BW) with 95% confidence intervals. Causes of the neonatal and infant deaths were categorized according to the International Statistical Classification of Diseases 10th version, and population attributable fractions of GA and BW strata were analyzed. Perinatal risks of infant mortalities in continuum periods were estimated by Cox regression models.

**Results:**

Among the whole livebirth population (59056), 7960 were hospitalized (prevalence 13.5%), with 168 (2.8‰) in-hospital deaths. The NMR was 3.6 (3.2, 4.1)‰ and IMR 4.9 (1.4, 4.5)‰, with additionally 35 (0.6‰) deaths at delivery. The major causes of infant deaths were perinatal conditions (2.6‰, mainly preterm-related), congenital anomalies (1.5‰), sudden unexpected death in infancy (0.6‰) and other causes (0.2‰). The deaths caused by preterm and low BW (LBW) accounted for 50% and 40% of NMR and IMR, with 20-30% contributed by EPT or extremely LBW, respectively. Multivariable Cox regression analysis revealed that peripartum factors and LBW strata had strong association with early- and late-neonatal deaths, whereas those of GA < 28 weeks were highly associated with postneonatal deaths. Congenital anomalies and neonatal hospitalization remained high death risks over the entire infancy, whereas maternal co-morbidities/complications were modestly associated with neonatal but not postneonatal infant mortality.

**Conclusions:**

The NMR, IMR, major causes of deaths and associated perinatal risks in continuum periods of infancy, denote the status and quality improvement of the regional perinatal-neonatal care associated with socioeconomic development. The study concept, applicability and representativeness may be validated in other evolving regions or countries for genuine comparison and better maternal-infant healthcare.

**Supplementary Information:**

The online version contains supplementary material available at 10.1186/s12884-022-04653-8.

## Background

As China has been dramatically progressing in maternal and child healthcare in the past decades with growing economy and social welfare in transition [1–3], the under 5 mortality rate (per 1000 livebirths) has declined steadily from 53.7 in 1990 to 8.6 in 2019, with infant mortality rate (IMR) from 42.1 to 6.8, and neonatal mortality rate (NMR) from 29.5 to 3.9 [4], respectively. The foremost achievement is the substantial reduction of maternal and infant death of perinatal causes by early surveillance for high-risk pregnancy, centralized hospital delivery, prevention of preterm birth, and early postnatal care of critically ill infants [1–3, 5]. All these are contributed by the improved perinatal-neonatal care infrastructure and universal health insurance policy in effect since 2010 [1, 5].

Currently reported epidemiological data of NMR and IMR in China are derived from either selective hospital birth registries or sampling of nationwide birth surveillance, the Maternal and Child Health Surveillance System (MCHSS) [1–3, 6, 7], for making maternal-infant health policies [3, 7]. However, these data sources have limited function in exploring causation of perinatal-neonatal care and outcome due to stratified sampling of data collection, incompleteness and inconsistency of definitions for informative data, and exclusion of births below 28 weeks of gestational age (GA) [1–3, 6, 7]. It results in altered source of population and difficulty for comparison of vital statistics especially in very or extremely preterm birth in reference to the relevant international reports [8–10]. Besides, the lack of integrated information of maternal/pregnancy factors, birth status and in-hospital care of neonates hinders the exploration of risk factors and their impact, as contributing coefficient and probability through regression models, on adverse outcome associated with major morbidities leading to neonatal and infant deaths [1–3, 6, 7]. By now, there are rare epidemiological studies towards perinatal, neonatal and infant outcomes with comprehensive antenatal, peripartum and postnatal information based on a whole regional livebirth population in China. It might also restrain the evaluation of efficiencies of perinatal-neonatal and infant healthcare systems under local socioeconomic status (SES) and medical resource settings.

In 2010 and 2015, we conducted two prospective studies of perinatal-neonatal outcome assessment, based on complete birth population in Huai’an, through unified definitions and inclusion of almost all births under 28 weeks as extreme preterm [11–14]. With these efforts, we attempted to provide comprehensive vital statistic data on perinatal and neonatal mortality rates, with estimation of perinatal risks and outcome based on whole birth population from a sub-provincial (prefectural) region. The source population constituted approximately 0.4% of the annually nationwide livebirth population (15 million in 2015), and likely represented contemporaneous perinatal-neonatal care in certain proportion of sub-provincial regions and population as well as SES in China. From the 2015 study [13, 14], we moved forward to focus on detailed information of neonatal hospitalization and long-term outcomes of survival through infancy. We assumed that both IMR and NMR and potentially causal relations associated with perinatal risks may be derived, that the reliability and applicability of methodology be tested as a paradigm in vital statistics. We aimed to construct livebirth population-based norm of survival in analyzing regional NMR, IMR and associated relative risks of perinatal origin for genuine comparison with international data sources.

## Methods

### Study population, data sources and ethics approval

**Fig. 1:**
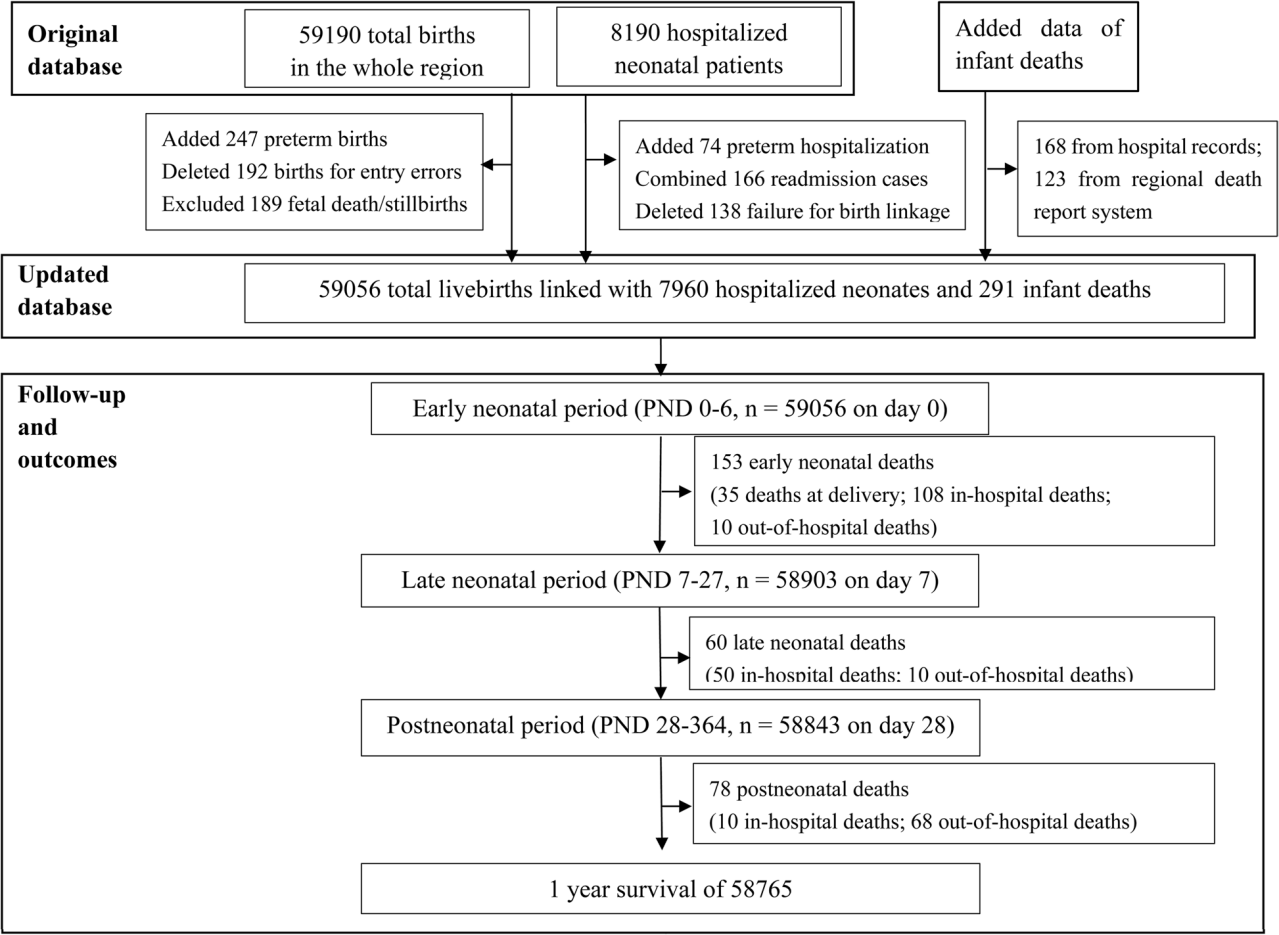
Flow chart describing the selection and follow-up of the updated cohort population. PND, postnatal days

The source population included the whole regional livebirths with maternal and neonatal information from all obstetrical departments, neonatal wards and/or neonatal intensive care units (NICU) in Huai’an, by prospective data collection from 1 January to 31 December in 2015 [13, 14]. To obtain death information of all liveborn infants, we retrieved all death reports in the original birth database to identify the major causes of deaths of livebirths at delivery, as well as all hospitalized neonatal case records by redefining causes of in-hospital deaths. For those infant deaths without admission into, or after discharge from, any neonatal ward or NICU were classified as out-of-hospital deaths, which were derived from regional death report system (MCHSS) after verification of corresponding clinical records. By integrating the data of all infant deaths from the two sources, a new integrated database was established as a cohort linking information of livebirths, neonatal hospitalization and/or infant death for final analysis [15]. Works of retrospective data collection and analysis were conducted in 2018-2021. A study flow chart of the cohort linkage, data of infants excluded and included are shown in Fig. 1.

The study protocol was approved by the ethics committee of Children’s Hospital of Fudan University, and adopted by the Huai’an Women and Children’s Hospital and all other participating hospitals in Huai’an [13–15]. As no specific intervention was applied, informed consent from parents/guardians was waived.

### Definitions of variables

Definitions regarding vital statistics were based on the original survey [13, 14] and diagnoses of any diseases were defined and categorized according to established clinical diagnostic criteria and the International Statistical Classification of Diseases and Related Health Problems, 10th Revision (ICD-10) [16]. Briefly, livebirth was defined as birth presenting with any signs of life, such as breaths, heart beating, pulsation of the umbilical cord, or definite movement of voluntary muscles [16]. Perinatal variables included maternal and neonatal demographics and medical conditions. Maternal demographic characteristics and SES such as maternal age, urban or rural residency, educational level and prenatal care were included. Inadequate prenatal care was defined as fewer than six visits (half of the recommended average times) to any prenatal care facility during the pregnancy [17]. In addition, major co-morbidities/complications of pregnancy were included such as hypertensive disorder of pregnancy (HDP), gestational diabetes of mellitus (GDM), moderate-to-severe anemia, prelabor rupture of membrane (PROM), abnormalities of placenta and umbilical cord, antenatal steroids, multiple births, amniotic fluid (AF) contamination, fetal distress and mode of delivery [14]. As for neonatal variables, GA was classified with 6 subgroups: 25^+ 0^- 27^+ 6^ weeks (extreme preterm or EPT, no livebirths in GA < 25 weeks), 28^+ 0^-31^+ 6^ weeks (very preterm, VPT), 32^+ 0^-36^+ 6^ weeks (moderate-late preterm), 37^+ 0^-38^+ 6^ weeks (early term), 39^+ 0^-41^+ 6^ weeks (full term), > 42^+ 0^ weeks (post-term); and birthweight (BW) with 5 subgroups: < 1000 g (extremely low BW, ELBW), 1000-1499 g (very low BW, VLBW), 1500-2499 g (low BW), 2500-3999 g (normal BW), > 4000 g (macrosomia) [16]. GA < 37 weeks and BW < 2500 g were defined as preterm and LBW, respectively. Other variables for neonatal status and exposed risks included sex, small for GA (SGA), resuscitation at delivery room (DR), Apgar score, congenital anomalies, and neonatal hospitalization. The need for in-hospital care was defined as admissions into any neonatal ward or NICU within neonatal period (0-27 postnatal days) for term births or within adjusted GA of preterm infants equivalent to 28 postnatal days for term births. All the above-mentioned perinatal and neonatal diagnoses or definitions were consistent with previous studies [11–15].

As for the cohort outcome, neonatal mortality as deaths within 28 postnatal days (PND) and infant mortality was defined as deaths during infancy (0-364 PND). Besides, infant deaths were classified into three periods: 0-6 days as early neonatal deaths (including deaths at delivery); 7-27 days as late neonatal deaths; and 28-364 days as postneonatal deaths. The causes of death were classified into 4 main categories with respective codes following ICD-10 guidelines: (1) perinatal conditions, P00-P96; (2) congenital anomalies, Q00-Q99; (3) sudden unexpected death in infancy (SUDI), V01-Y89 and R00-R99; and (4) other causes, all other codes [16, 18].

### Statistical analysis

Statistical analysis was performed using SPSS 23.0 (IBM, Chicago, IL), with figures produced by GraphPad PRISM 6.02 (GraphPad Software, La Jolla, CA). Data are presented as number (n) and rate in percent (%) or per thousand (‰). For descriptive analysis, we compared basic characteristics between the cohorts with or without neonatal hospitalization in those of preterm or non-preterm (term and post-term) with Pearson Chi-square test or Fisher exact test for group comparison, as well as of all livebirths with the adjustment of preterm by Cochran-Mantel-Haenszel tests. The total NMR and IMR were calculated by the number of deaths referring to total livebirths with further analyzed by GA or BW strata as well as cause-specific classification. The 95% confidence interval (CI) of rate was estimated by a null multilevel Poisson model with empirical, robust standard errors but no explanatory variables [19]. GA or BW stratified survival during neonatal and postneonatal periods was determined using Kaplan-Meier survival analysis.

The relative risk (RR) and 95% CI of GA and BW for neonatal and infant mortality were estimated by a generalized linear model of Poisson regression with robust variance [19, 20], with GA 39-41 weeks and BW 2500-3999 g as reference, respectively. Population attributable fraction (PAF) of each subgroups was calculated as: *PAF*_*i*_ = *p*_i_*(*RR*_*i*_ - 1)/[1 + Σ*n i = 1p*_i_*(*RR*_*i*_ - 1)]. RR_i_ and p_i_ respectively represented relative risk and prevalence in the target population for the i^th^ level of risk factors, with n as the total number of exposure levels [21]. This equation evaluated the proportional reduction expected in the subgroup individuals if the known level of risk factors were eliminated from the target population. An example of this calculation was shown in additional file 1.

Uni- and multivariable Cox regression models were used to examine the hazard ratio (HR) and 95%CI of perinatal risks associated with infant mortality. Perinatal risks included all the maternal and neonatal factors mentioned above. Selected adjustments were based on the final step of multivariable regression model with backward stepwise selection. Since the proportionality assumption was violated in the infant mortality analyses, the follow-up time was also split into early-, late-neonatal and postneonatal periods as defined above. All the survivals at the initial days of each period entered the cohort and were analyzed. The validity of the proportionality assumptions of the Cox model of deaths in these three periods was evaluated by testing Schoenfield residuals [22, 23]. *P* < 0.05 was considered statistically significant.

## Results

### Descriptive analysis for livebirth cohort

In the current cohort of the whole regional birth population, 59,056 livebirths were included (by excluding 189 stillbirths). There were 213 neonatal and 291 infant deaths, with the livebirth prevalence of NMR and IMR were 3.6‰ (95% CI 3.2‰, 4.1‰), and 4.9‰ (4.4‰, 5.5‰), respectively. Among all livebirths, 35 died at delivery with the incidence of 0.6‰ (0.4‰, 0.8‰). The incidence rates of preterm and LBW were 4.3% (4.1%, 4.4%) and 3.1% (3.0%, 3.3%), respectively. Totally, 7960 neonates were hospitalized [13.5% (13.2%, 13.8%)] and 168 [2.8‰ (2.5‰, 3.3‰)] died during their hospitalization, which contributed to 57.7% of the total infant deaths (Table 1).

**Table 1:** Perinatal demographic status, prevalence and mortality rates of preterm and non-preterm cohorts with or without hospitalization of all livebirths

	Preterm		Non-Preterm^**a**^					
	Hospitalization^**b**^	Non-hospitalization	Hospitalization^**b**^	Non-hospitalization	Total	***P***_***1***_	***P***_***2***_	***P***_***3***_
Total livebirths^c^	1941 (3.3)	573 (1.0)	6019 (10.2)	50,523 (85.6)	59,056			
Maternal age, y								
< 20	74 (3.8)	23 (4.0)	159 (2.6)	1706 (3.4)	1962 (3.3)	0.826	0.003	0.034
20-34	1637 (84.3)	496 (86.6)	5377 (89.3)	44,523 (88.1)	52,033 (88.1)	0.192	0.006	0.982
> 35	230 (11.8)	54 (9.4)	483 (8.0)	4294 (8.5)	5061 (8.6)	0.107	0.211	0.184
Rural residency	1135 (58.5)	342 (59.7)	3681 (61.2)	29,331 (58.1)	34,489 (58.4)	0.605	< 0.001	< 0.001
Education > 9 y	846 (43.6)	290 (50.6)	2074 (34.5)	19,850 (39.3)	23,060 (39.1)	0.003	< 0.001	< 0.001
Inadequate prenatal care	1011 (52.1)	239 (41.7)	3078 (51.1)	20,780 (41.1)	25,108 (42.5)	< 0.001	< 0.001	< 0.001
Multipara	1171 (60.3)	370 (64.6)	3441 (57.2)	30,349 (60.1)	35,331 (59.8)	0.067	< 0.001	< 0.001
HDP	382 (19.7)	82 (14.3)	399 (6.6)	2030 (4.0)	2893 (4.9)	0.004	< 0.001	< 0.001
GDM	100 (5.2)	29 (5.1)	150 (2.5)	624 (1.2)	903 (1.5)	0.931	< 0.001	< 0.001
Anemia	247 (12.7)	66 (11.5)	928 (15.4)	3209 (6.4)	4450 (7.5)	0.442	< 0.001	< 0.001
PROM	660 (34.0)	160 (27.9)	631 (10.5)	3775 (7.5)	5226 (8.6)	0.006	< 0.001	< 0.001
Placenta	198 (10.2)	26 (4.5)	149 (2.5)	535 (1.1)	908 (1.5)	< 0.001	< 0.001	< 0.001
Umbilical cord	162 (8.3)	13 (2.3)	522 (8.7)	1370 (2.7)	2067 (3.5)	< 0.001	< 0.001	< 0.001
Antenatal steroids	908 (46.8)	173 (30.2)	29 (0.5)	44 (0.1)	1154 (2.0)	< 0.001	< 0.001	< 0.001
Fetal distress	23 (1.2)	9 (1.6)	82 (1.4)	572 (1.1)	686 (1.2)	0.469	0.114	0.159
Cesarean delivery	1169 (60.2)	340 (59.3)	2996 (49.8)	27,538 (54.5)	32,043 (54.3)	0.702	< 0.001	< 0.001
AF contamination	154 (7.9)	37 (6.5)	805 (13.4)	6635 (13.1)	7631 (12.9)	0.241	0.600	0.012
Multiple births	390 (20.1)	150 (26.2)	159 (2.6)	560 (1.1)	1259 (2.1)	0.002	< 0.001	< 0.001
GA 25-27 (week)	40 (2.1)	14 (2.4)	–	–	54 (0.1)	0.579	–	–
28-31	241 (12.4)	24 (4.2)	–	–	265 (0.5)	< 0.001	–	–
32-36	1660 (85.5)	535 (93.4)	–	–	2195 (3.7)	< 0.001	–	–
37-38	–	–	1900 (31.6)	11,016 (21.8)	12,916 (21.9)	–	< 0.001	–
39-41	–	–	4076 (67.7)	38,922 (77)	42,998 (72.8)	–	< 0.001	–
> 42	–	–	43 (0.7)	585 (1.2)	628 (1.1)	–	0.002	–
BW < 1000 (g)	20 (1.0)	14 (2.4)	0	0	34 (0.1)	0.010	–	–
1000-1499	177 (9.1)	15 (2.6)	6 (0.1)	14 (0.03)	212 (0.4)	< 0.001	0.050	< 0.001
1500-2499	942 (48.5)	128 (22.3)	213 (3.5)	319 (0.6)	1602 (2.7)	< 0.001	< 0.001	< 0.001
2500-3999	785 (40.4)	406 (70.9)	5064 (84.1)	42,851 (84.8)	49,106 (83.2)	< 0.001	0.165	< 0.001
> 4000	17 (0.9)	10 (1.7)	736 (12.2)	7339 (14.5)	8102 (13.7)	0.076	< 0.001	< 0.001
Low BW	1139 (58.7)	157 (27.4)	219 (3.6)	333 (0.7)	1848 (3.1)	< 0.001	< 0.001	< 0.001
SGA	103 (5.3)	23 (4.0)	311 (5.2)	2084 (4.1)	2521 (4.3)	0.213	< 0.001	< 0.001
Male	1088 (56.1)	329 (57.4)	3415 (56.7)	26,750 (52.9)	31,582 (53.5)	0.563	< 0.001	< 0.001
Apgar 1 min < 7	575 (29.6)	65 (11.3)	571 (9.5)	246 (0.5)	1457 (2.5)	< 0.001	< 0.001	< 0.001
Apgar 5 min < 7	167 (8.6)	36 (6.3)	164 (2.7)	57 (0.1)	424 (0.7)	0.073	< 0.001	< 0.001
DR resuscitation	347 (17.9)	88 (15.4)	270 (4.5)	1193 (2.4)	1898 (3.2)	0.161	< 0.001	< 0.001
Intubation	21 (1.1)	4 (0.7)	30 (0.5)	58 (0.1)	113 (0.2)	0.416	< 0.001	< 0.001
Chest compression	32 (1.6)	5 (0.9)	31 (0.5)	36 (0.1)	104 (0.2)	0.175	< 0.001	< 0.001
Adrenaline	14 (0.7)	1 (0.2)	20 (0.3)	9 (0.02)	44 (0.1)	0.135	< 0.001	< 0.001
Congenital anomalies	159 (8.2)	14 (2.4)	413 (6.9)	204 (0.4)	790 (1.3)	< 0.001	< 0.001	< 0.001
In-hospital mortality^d^	89 (4.6)	–	79 (1.3)	–	168 (0.3)	–	–	–
Out-of-hospital mortality^e^	4 (0.2)	3 (0.5)	30 (0.5)	51 (0.1)	88 (0.2)	0.012	0.014	0.004
Deaths at DR	–	27 (4.7)	–	8 (0.02)	35 (0.1)	–	–	–
Neonatal mortality^f^	82 (4.2)	27 (4.7)	84 (1.4)	20 (0.04)	213 (0.4)	0.615	< 0.001	< 0.001
0-6 PND (early)	55 (2.8)	27 (4.7)	55 (0.9)	16 (0.03)	153 (0.3)	0.026	< 0.001	< 0.001
7-27 PND (late)	27 (1.4)	0	29 (0.5)	4 (0.01)	60 (0.1)	0.005	< 0.001	< 0.001
Postneonatal mortality^g^	11 (0.6)	3 (0.5)	25 (0.4)	39 (0.1)	78 (0.1)	0.903	< 0.001	< 0.001
Infant mortality^h^	93 (4.8)	30 (5.2)	109 (1.8)	59 (0.1)	291 (0.5)	0.665	< 0.001	< 0.001

*Abbreviations: HDP* Hypertensive disorder of pregnancy, *GDM* Gestational diabetes of mellitus, *PROM* Prelabor rupture of membrane, *PROM* Prelabor rupture of membrane, *AF* Amniotic fluid, *BW* Birthweight, *GA* Gestational age, *SGA* Small for gestational age, *DR* Delivery room, *PND* Postnatal days

Values are given as n (%), referring to all livebirths in respective columns unless otherwise stated. *P* values are the probability between the cohorts of preterm (*P*_*1*_) and non-preterm (*P*_*2*_) livebirths with and without hospitalization by Pearson Chi-square tests or Fisher exact tests; or between the cohorts with and without hospitalization of all livebirths (*P*_*3*_) with the adjustment of preterm by Cochran-Mantel-Haenszel tests

^a^. Non-preterm denotes term and post-term

^b^. Hospitalization refers to admitted infants in any neonatal wards or neonatal intensive care units (NICU)

^c^. Rate (%) in parentheses refers to total livebirths (59056)

^d^. In-hospital mortality includes all deaths during hospitalization in any neonatal wards or NICU (excluding deaths at DR)

^e^. Out-of-hospital mortality includes infant deaths after discharged from, as well as deaths without admission to, neonatal ward or NICU during infant period (excluding deaths at DR)

^f^. Neonatal mortality includes deaths of livebirth during 0-27 PND (including deaths at DR)

^g^. Postneonatal mortality includes deaths of livebirth during 28-364 PND

^h^. Infant mortality includes all deaths of livebirth during 0-364 PND

Table 1 lists the basic information of perinatal characteristics and infant outcome among hospitalized and non-hospitalized neonates by preterm or non-preterm (term and post-term) strata. In comparison to non-preterm livebirths, those livebirths with GA < 37 weeks presented with higher rates of mothers with early or late childbearing, less rural residency and lower education level, as well as more co-morbidities/complications, such as HDP, GDP, PROM, abnormalities of placenta, antenatal steroids, and cesarean delivery, regardless of hospitalization (*P* < 0.05). Compared to those without hospitalization, hospitalized neonates generally presented higher prevalence of maternal co-morbidities/complications, LBW, low Apgar scores, rigorous DR resuscitation and congenital anomalies, especially in those of preterm (*P* < 0.05). As for the outcome, infants with neonatal hospitalization accounted for 69.4% (202/291) of total infant deaths, with 71.9% (110/153), 93.3% (56/60) and 46.2% (36/78) as early-, late-neonatal and postneonatal mortalities, respectively. For those without hospitalization, deaths of preterm infants were 90.0% occurred at delivery (27/30), but of term ones mainly at postneonatal period (39/59, 66.1%).

### GA and BW stratified NMR and IMR

Table 2 lists the stratified rates for NMR and IMR by GA and BW strata, with the detailed numbers of each week listed in Table S1 and BW in increment of 250 g in Table S2. There were 77.2% (1941/2514) of preterm and 73.5% (1358/1848) LBW hospitalized, with 81.5% and 84.0%, respectively, admitted on the first postnatal day. Since the large proportion of DR deaths existed in newborns of GA < 28 weeks (12/54, 22.2%) and BW < 1000 g (14/34, 41.2%), in-hospital rates of EPT (74.1%) and ELBW (58.8%) were relatively lower than other preterm and LBW strata (70-90%) (Table S1, S2). The IMR for preterm and LBW were 4.9% (123/2514) and 7.0% (129/1848) while for those of GA < 32 weeks and BW < 1500 g, 27.0% (86/319) and 31.7% (78/246), respectively, with > 80% occurred in the neonatal period.

**Table 2:** Neonatal and infant mortality risks and population attributable fraction (PAF) by gestational age and birthweight strata

	N	Rate, ‰ (95% CI)	RR (95% CI)	PAF (%)
**Neonatal deaths**	213	3.6 (3.2, 4.1)		
GA 25-27 (week)	28	518.5 (401.0, 670.5)	365.50 (255.23, 523.40)^***^	13.11
28-31	43	181.1 (140.2, 234.0)	127.68 (89.22, 182.70)^***^	22.35
32-36	33	15.0 (10.7, 21.1)	10.60 (6.95, 16.15)^***^	14.03
37-38	41	3.2 (2.3, 4.3)	2.24 (1.51, 3.32)^***^	10.66
39-41	61	1.4 (1.1, 1.8)	1.00 (Reference)	
> 42	2	3.2 (0.8, 12.7)	2.25 (0.55, 9.16)	0.52
BW < 1000 (g)	25	735.3 (601.0, 899.6)	392.47 (294.56, 522.92)^***^	11.70
1000-1499	44	207.5 (159.5, 270.0)	110.78 (79.41, 154.55)^***^	20.46
1500-2499	46	28.7 (21.6, 38.2)	15.33 (10.80, 21.76)	20.18
2500-3999	92	1.9 (1.5, 2.3)	1.00 (Reference)	
> 4000	6	0.7 (0.3, 1.6)	0.40 (0.17, 0.90)^*^	−4.27
**Infant deaths**	291	4.9 (4.4, 5.5)		
GA 25-27 (week)	34	629.6 (513.2, 772.5)	246.12 (186.59, 324.64)^***^	11.63
28-31	52	196.2 (153.8, 250.4)	76.70 (56.43, 104.26)^***^	17.62
32-36	37	16.9 (12.2, 23.2)	6.59 (4.55, 9.54)^***^	10.78
37-38	55	4.3 (3.3, 5.5)	1.67 (1.21, 2.30)^**^	7.60
39-41	110	2.6 (2.1, 3.1)	1.00 (Reference)	
> 42	3	4.8 (1.5, 14.8)	1.87 (0.60, 5.86)	0.48
BW < 1000 (g)	28	823.5 (704.9, 926.2)	271.41 (217.07, 339.36)^***^	9.59
1000-1499	50	235.8 (185.1, 300.5)	77.73 (58.13, 103.94)^***^	16.96
1500-2499	51	31.8 (24.3, 41.7)	10.49 (7.66, 14.36)^***^	15.85
2500-3999	149	3.0 (2.6, 3.6)	1.00 (Reference)	
> 4000	13	1.6 (0.9, 2.8)	0.53 (0.30, 0.93)^*^	−3.97

*Abbreviations: RR* Relative risk, in comparison with the subgroups in reference, *CI* confidence interval, *GA* Gestational age, *BW* Birthweight, *PAF* Population attributable fraction. * *P* < 0.05, ** *P* < 0.01, and *** *P* < 0.001 for comparisons with the reference group

Values are n or ratio (‰). Ratio refers to per thousand (‰) of livebirths at each stratum except the total number of neonatal or infant deaths which refers to total livebirths (59056)

Table 2 also demonstrates RR and PAF of neonatal and infant mortality by GA and BW strata. It showed that both preterm and LBW had higher risks of deaths, and contributed to a population level risk (PAF) of neonatal deaths of 50% and infant deaths of 40%, respectively. In both neonatal and infant periods, although the RR of EPT or ELBW were the highest (RR > 200), the small proportion constituted a relatively lower PAF (10%) than those of VPT and VLBW (RR > 50, PAF 15-25%). Notably, early term infants presented a PAF of 10.7% for NMR and 7.6% for IMR even with small RR, whereas macrosomia was associated with decreased death risk with PAF < 0.

**Fig. 2:**
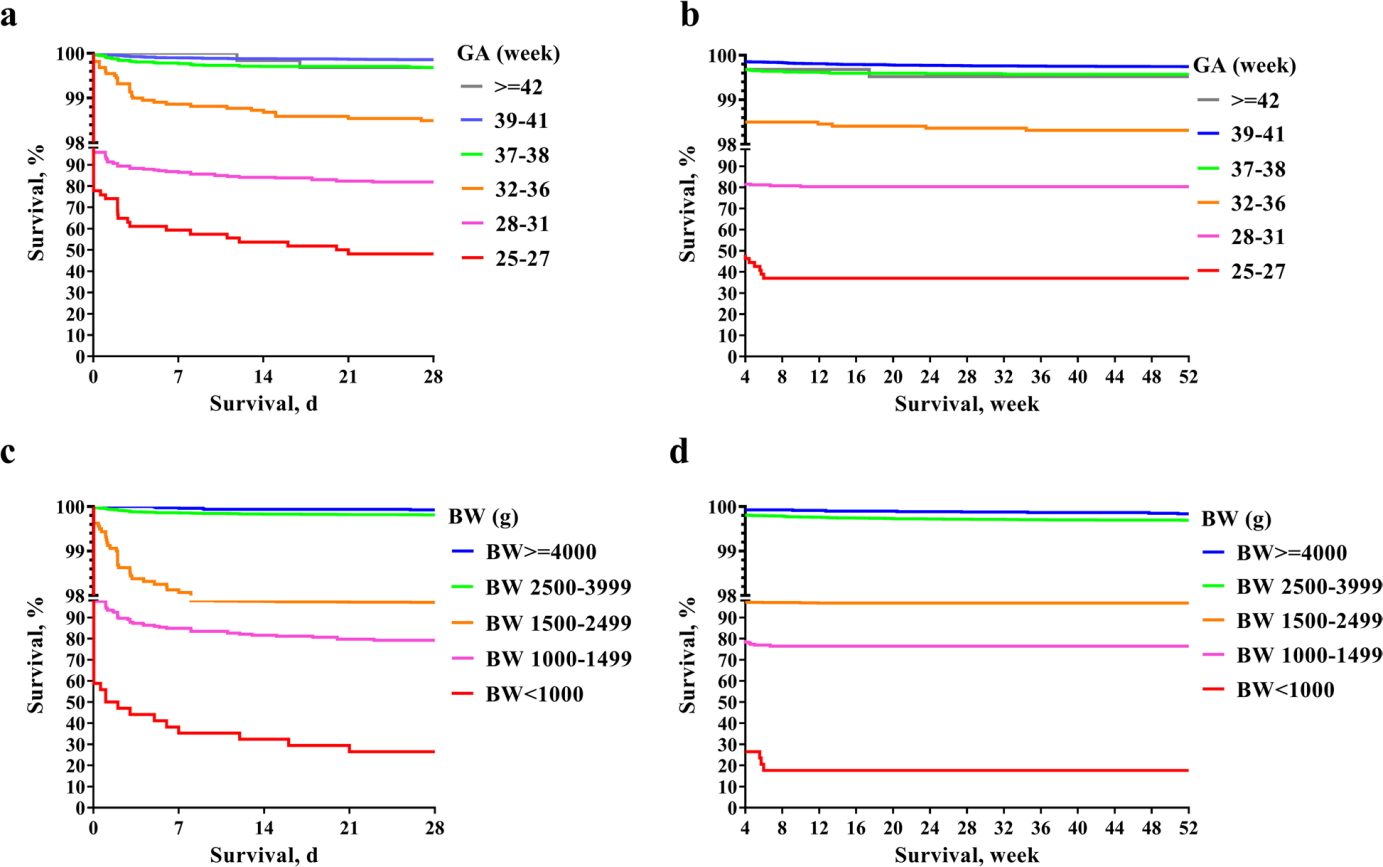
One-year survival of regional livebirths by gestational age (GA) or birthweight (BW) strata. a. Survival during first 28 days by GA strata; b. Survival during 28-364 postnatal days by GA strata; c. Survival during first 28 days by BW strata; d. Survival during 28-364 postnatal days by BW strata

Figure 2 shows the GA or BW stratified neonatal and postneonatal survival, which suggested that deaths within 28 PND accounted for large part (> 70%) of total infant deaths. The 50% of one-year survival rates of all livebirths were at GA 25-27 weeks and BW < 1000 g, while the total survival rates for those with GA 28-31 weeks and BW 1500-1499 g were > 75%, and > 95% for those with GA > 32 weeks and BW > 1500 g.

### Causes of neonatal and infant mortality

**Fig. 3:**
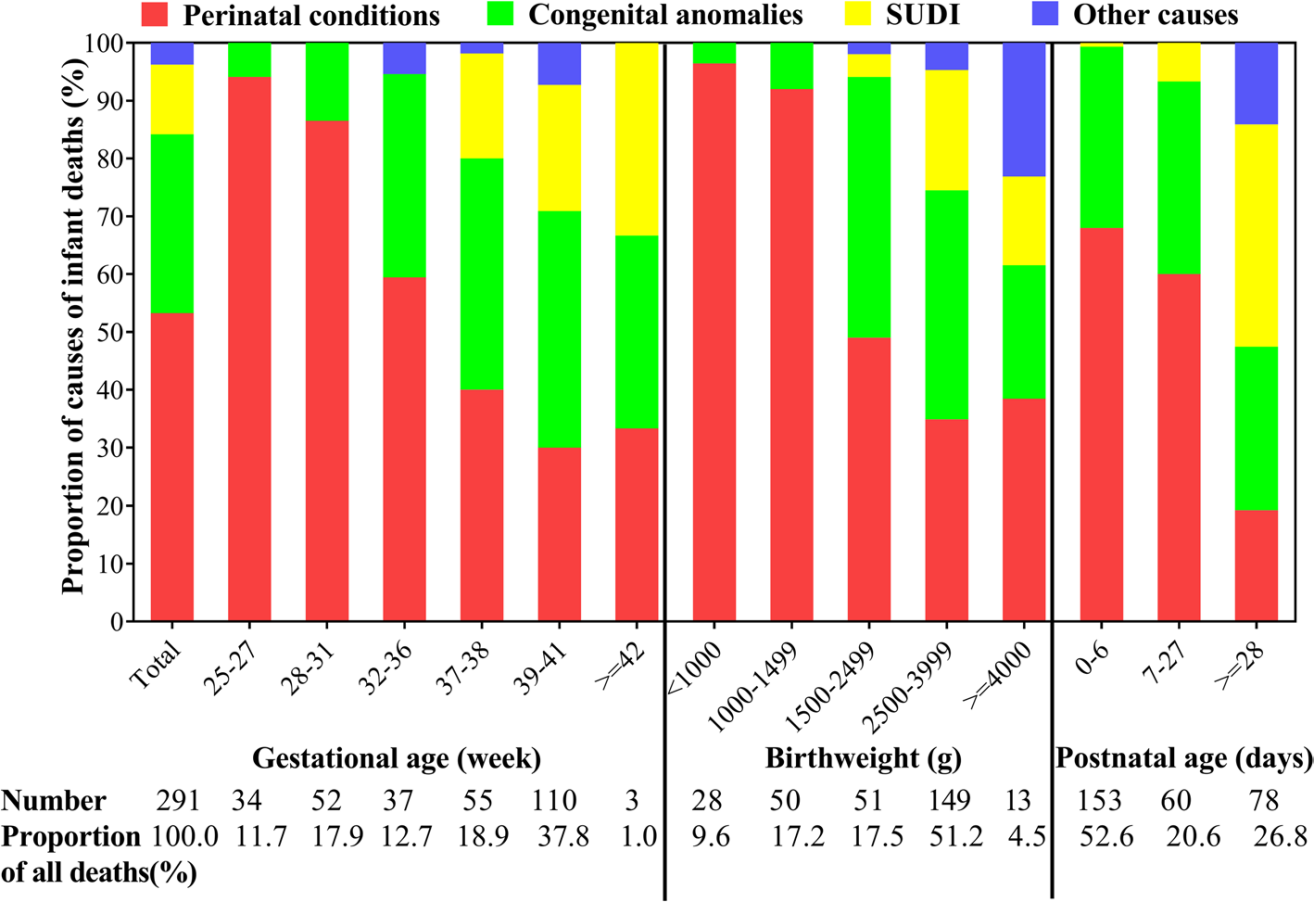
Proportional mortality for major causes of infant death by gestational age, birthweight and postnatal age. SUDI, sudden unexpected death in infancy

As Table 3 and Fig. 3 shown, the major cause categorized IMR were perinatal conditions (2.6‰), congenital anomalies (1.5‰), SUDI (0.6‰) and other causes (0.2‰). Perinatal conditions contributed to 65.7% NMR and 53.3% IMR while that for congenital anomalies, 31.9% and 30.9%, respectively. In preterm and LBW deaths, perinatal conditions accounted for 80.5% (99/123) and 76.0% (98/129) IMR, respectively, especially in those with GA < 32 weeks (77/86, 89.5%) and BW < 1500 g (73/78, 93.6%). The proportion of congenital anomalies increased with GA and BW advancing. The deaths due to SUDI and other causes, though very few, occurred mainly in term and normal BW infants and postneonatal period. Out-of-hospital deaths occurred mainly in term, regular BW and postneonatal period, together with that of SUDI and congenital anomalies being the main causes of infant deaths (Table S3).

**Table 3:** The prevalence of cause-specific neonatal and infant mortality rate of all livebirths

	Neonatal death		Infant death	
	n	Rate, ‰ (95%CI)	n	Rate, ‰ (95%CI)
Perinatal conditions	140	2.4 (2.0, 2.8)	155	2.6 (2.2, 3.1)
Congenital anomalies	68	1.2 (0.9, 1.5)	90	1.5 (1.2, 1.9)
SUDI	5	0.1 (0.03, 0.2)	35	0.6 (0.4, 0.8)
Other causes	0	0	11	0.2 (0.1, 0.3)

*Abbreviations: CI* Confidence interval, *SUDI* Sudden unexpected death in infancy

Values are n or ratio (‰). Ratio refers to per thousand (‰) of total livebirths (59056)

### Multivariable adjusted perinatal risks

The multivariable Cox regression analyses of adjusted HR (aHR) of perinatal risks associated with infant mortality over different periods are shown in Table 4 (with univariable analyses listed in Table S4). In early neonatal period, BW < 1000 g and low Apgar score were associated with high risks (aHRs > 10) for deaths, while moderate risks (aHRs 3-5) were found in GA < 32 weeks, BW 1000-1499 g, congenital anomalies and neonatal hospitalization. Both maternal high education level and antenatal steroids markedly lowered the risks for early neonatal deaths, whereas abnormality of umbilical cord and AF contamination had increased risks (aHRs 2). In late neonatal period, death risks were strongly associated with BW < 1500 g, GA > 42 weeks, congenital anomalies and neonatal hospitalization (aHRs ranged from 9 to 90). In postneonatal period, aHR of GA < 28 weeks was as high as 33.31 (12.23, 90.72), whereas BW in strata contributed less significantly to the death hence being removed from the multivariable model. Maternal education > 9 years was also associated with lower risks with aHR of 0.59 (0.36, 0.97). A moderate-to-strong association of congenital anomalies [aHR 6.90 (3.61, 13.19)] and neonatal hospitalization [aHR 3.30 (1.91, 5.71)] with death was also shown in posteneonatal period. SGA was only significantly associated with early neonatal death in the univariable regression analysis (HR 2.81), but not associated with either neonatal or infant death in the multivariable analysis.

**Table 4:** Multivariable analysis of perinatal risk factors for infant mortality at different periods by Cox regression model

	Adjusted^**a**^ HR (95% CI)		
	Early neonatal death	Late neonatal death	Postneonatal death
GA 25-27 (week)	2.99 (1.10, 8.16)^*^	0.59 (0.09, 3.81)	33.31 (12.23, 90.72)^***^
28-31	3.62 (1.53, 8.58)^**^	1.02 (0.29, 3.66)	2.31 (0.72, 7.41)
32-36	2.15 (1.08, 4.27)^*^	0.34 (0.12, 1.02)	0.39 (0.13, 1.19)
37-38	1.62 (1.00, 2.65)	1.34 (0.64, 2.81)	0.72 (0.39, 1.32)
39-41	1.00 (Reference)	1.00 (Reference)	1.00 (Reference)
> 42	–	9.77 (2.24, 42.69)^**^	1.52 (0.21, 11.10)
BW < 1000 (g)	27.15 (10.36, 71.16)^***^	86.88 (12.37, 610.07)^***^	NI
1000-1499	4.26 (1.85, 9.79)^**^	9.08 (2.48, 33.24)^**^	NI
1500-2499	2.02 (1.08, 3.78)^*^	4.76 (2.02, 11.24)^***^	NI
2500-3999	1.00 (Reference)	1.00 (Reference)	NI
> 4000	0.19 (0.05, 0.79)^*^	1.17 (0.40, 3.41)	NI
Maternal age < 20 (y)	NI	2.54 (0.90, 7.21)	NI
20-34	NI	1.00 (Reference)	NI
> 35	NI	2.32 (1.18, 4.58)^*^	NI
Rural residency	1.41 (0.96, 2.09)	NI	NI
Education > 9 y	0.66 (0.45, 0.96)^*^	NI	0.59 (0.36, 0.97)^*^
PROM	NI	1.89 (1.02, 3.51)^*^	NI
Umbilical cord	1.82 (1.07, 3.08)^*^	NI	NI
Antenatal steroids	0.42 (0.26, 0.68)^***^	NI	NI
AF contamination	2.03 (1.36, 3.01)^***^	2.00 (1.07, 3.73)^*^	NI
Multiple births	NI	NI	4.31 (2.02, 9.23)^***^
Apgar 5-min < 7	17.89 (11.40, 28.07)^***^	1.90 (0.92, 3.92)	NI
Congenital anomalies	3.18 (2.13, 4.75)^***^	12.07 (6.85, 21.27)^***^	6.90 (3.61, 13.19)^***^
Hospitalization	3.03 (1.92, 4.80)^***^	31.41 (10.64, 92.67)^***^	3.30 (1.91, 5.71)^***^

*Abbreviations: HR* Hazard ratio, in comparison with the subgroups in reference, *CI* Confidence interval, *GA* Gestational age, *BW* Birthweight, *PROM* Prelabor rupture of membrane, *AF* Amniotic fluid, *NI* Not included. * *P* < 0.05, ** *P* < 0.01, and *** *P* < 0.001 for comparisons with the reference group

^a^. Adjusted by all evaluated perinatal factors listed in the table but not those variables presenting as NI, i.e. not included in the final step of multivariable Cox regression model with backward stepwise selection

## Discussion

This cohort study reported the neonatal and infant mortality rates based on all livebirths and hospitalized neonatal population in Huai’an in 2015. The major findings revealed the survival outcome of all hospitalized livebirths along with those non-hospitalized. It showed the regional NMR and IMR with further stratification by GA and BW, and the major causes of infant deaths as perinatal conditions and congenital anomalies in the target population. Perinatal risks associated with the mortalities over whole infancy period were low GA and BW, congenital anomalies and neonatal hospitalization, whereas maternal SES and pregnancy co-morbidities/complications presented mild risks. This is an important progression in that the potentially causal relations between infant mortality and perinatal risks were clearly depicted. Our effort to the establishment of concept and methodology for the current data file should be complementary to the previous achievements through nationwide campaign [1–3, 6, 7, 11–14].

### Representativeness of the source populations

Huai’an is an emerging rural prefectural region of Jiangsu province, with a population of 5.6 million, approximately 0.4% of the total national population (1.37 billion), 56.1% urban residents, gross domestic production (GDP) of 274.5 billion Chinese Yuan (CNY, 6.2 = 1 USD), contributing to approximately 0.4% of total national GDP (67.67 trillion CNY) in 2015 [24, 25]. The average GDP per capita at 56,575 CNY in Huai’an was modestly above the corresponding national average (49,228 CNY), or in the third quartile range [24, 25]. Based on this SES background, we managed to have set up the linkage of livebirths and hospitalized neonatal data with perinatal information as source population basis for the infant outcomes in this whole sub-provincial region. As reported from this survey, the NMR and IMR in Huai’an were 3.6‰ and 4.9‰, whereas the nationally reported corresponding data were 5.4‰ and 8.1‰, respectively, in 2015 [24]. As there should exist large regional variations in China, our data file should have minimized over- or underestimation of the real status due to hospital sampling, lower efficient care standard, criteria of vital statistics excluding GA < 28 weeks births, or inaccurate livebirth counting at delivery, among other bias and confounding factors [1–3, 6–7]. Our previous participations of nationwide multi-center studies on the management and outcome of neonatal respiratory failure verified that the neonatal care level of Huai’an was above the national average [26–28], taken the regional SES into account [24, 25]. We thereby assumed that the regional maternal-infant healthcare, as reflected by the neonatal and infant outcome, may represent up to 20% of the sub-provincial (prefectural) regions, or 25% of the population, in other words, standing at the third quartile of SES and maternal-infant healthcare level of the country, mainly in the eastern, coastal provinces [2, 6, 24].

### Neonatal care in regional perinatal-neonatal network infrastructure

In comparison with the 2010 study, the hospitalized rate and in-hospital deaths rate in Huai’an were both higher in 2015 (13.5% vs. 11.3%, 2.8‰ vs. 2.4‰) [11]. These were associated with more centralized hospital delivery, adequate prenatal care for high-risk pregnancy, increased preterm livebirths [11–14]. It highlighted the progress of regional maternal and child healthcare coupling with the implementation of universal health insurance for maternal-infant healthcare since 2010 as well as the substantial SES advancement [24, 25]. In the current study, neonatal hospitalization was associated with maternal co-morbidities/complications, but marked associations were found with neonatal factors of moderate-to-severe morbidities such as being preterm birth, LBW, low Apgar scores, rigorous DR resuscitation and congenital anomalies. It indicated the efficiency of perinatal-neonatal healthcare system in the management of very and critically ill neonates as the most very and extremely preterm births were vigorously handled at birth and adequately hospitalized. Such information is not available from hospital admission-based sample population for neonatal and infant outcome in China [29, 30]. This study’s methodology and outcome measurements may serve as a paradigm for other evolving regions and countries to evaluate inter-regional or inter-institutional perinatal-neonatal healthcare efficiency and quality improvement.

### Causes-specific infant mortalities

As for the causes of infant death, perinatal risks and congenital anomalies accounted for 80% (mainly preterm and term infants, respectively). Prevalence of congenital anomalies differed in regions since it was closely related to SES and maternal status as well as diagnostic capacity [11, 13, 31, 32]. In this study, the prevalence of congenital anomalies was 13.4 per 1000 livebirths which was doubled from 6.8 per 1000 births in 2010 [11], a difference due to the inclusion of those diagnosed during hospitalization beyond first postnatal week or not [11, 12]. Although there was an apparently upward trend, it was still lower than financially well-off regions [31, 33] when the higher mortality rates remained [34, 35]. It is imperative to have a unified definition for reporting birth, livebirth, perinatal or hospitalization related birth defects or congenital anomalies, in order to estimate prevalence and causation of NMR and IMR in the context of prenatal screening and precise diagnostic process postnatally through early infancy [36]. The relative burden of mortality due to SUDI was a profound challenge in postneonatal infancy, calling for proper parental training through enhanced community-based, family-centered, maternal-infant healthcare.

### Perinatal risks associated with neonatal and infant mortality

The fact that maternal low education level and rural residency had higher risks of neonatal and postneonatal mortalities reflected the impact of SES on parental attitude towards critically ill newborns and financial support for ongoing medical care [11–14, 22]. In contrast, maternal morbidities only had modest impact on neonatal, but not the postneonatal, infant deaths. Our previous studies also demonstrated that high-risk pregnancy had an ambiguous association with perinatal mortality [12, 14]. Even in well developed countries, severe maternal morbidities conferred increased risks of infant mortality [RR 2.93 (2.51, 3.41)] [37]. In-depth analyses are needed to explore the severity of maternal morbidities in relation to infant outcome in resource-limited, evolving regions and countries.

Unlike the aforementioned maternal risk factors, neonatal morbidities had more profound and persistent effects on livebirth outcome. As reported, severe neonatal morbidities still exerted 3-4 times higher risks for postneonatal to under-5 mortalities [23] and increased need for rehospitalization between 1 and 6 years of age [38]. Recently, a large cohort study in Sweden also suggested that perinatal factors such as moderate or late preterm, SGA, malformations, low Apgar scores and admission to neonatal care were all associated with long-term neurodevelopmental disorder [39]. In the current study, preterm and LBW attributed to as high as 40-50% population level risks to neonatal and infant deaths. Moreover, early term infants still exerted 8-10% PAF even with small RR of deaths. Despite that those with GA < 32 weeks and BW < 1500 g presented with > 70% survival rates, lower GA and BW remained potent predictors for both neonatal and postneonatal mortalities as expected. To be noted, both neonatal and infant survival rates for livebirths of EPT were about 40-50%, and 20-30% of ELBW in current study, which were significantly lower than 80-90% reported by developed countries [9, 10, 40]. The highest RR and HR of EPT/ELBW for neonatal and infant death also indicated that the survival improvements for extremely premature infants remained the big challenge for local perinatal-neonatal care system. It is intriguing that SGA was not a risk for either neonatal or infant death in our multivariable Cox regression models. In general, SGA (< 3rd percentile) may be modestly associated with increased risks by 1-2 times in both neonatal and postneonatal mortality [41, 42]. However, recent study based on the whole livebirths in US and Norway reported that SGA was poorly predictive of neonatal mortality and cerebral palsy, while GA and BW predicted well [43]. The inconsistent definitions of SGA, and the inclusion of GA and BW in the adjusted model might contribute to the variations.

### Strengths and limitations

This study carried out a comprehensive analysis of outcome of livebirth, neonatal hospitalization and perinatal risks associated with neonatal and infant mortalities depicted in the early-, late-neonatal and postneonatal periods of infancy. With the PAF as part of the datafile, population-level prevalence and risks of neonatal and infant death in GA and BW categories were derived, which should be served as a quantitative indicator for inter-regional comparisons of the care efficiency of those with small GA or BW. The four major categories of causes of all infant death recommended by WHO ICD-10 were simple and convenient to operate. The source population was representative with regard to SES and perinatal-neonatal care standard, which may facilitate the extrapolation of the findings and serve as a benchmark for future study to validate.

Although we managed to minimize bias by scrutinizing clinical case records, retrospective linkage of database might be insufficient to the quality assurance in terms of accuracy and completeness. Even so, the proportion of unlinked livebirths was low (< 1%) and the linkage of all deaths was optimally managed. Besides, the consistence of data sources and underlying diseases for those out-of-hospital deaths might be influenced due to insufficient information of postneonatal hospitalization. However, with the unified morbidity and mortality definitions, analysis of the specific causes of in-hospital deaths should be applicable. Finally, variations of clinical practice standard across the network hospitals might exist under current cause categories of infant deaths. While its representativeness as source population and potentially causal relations of the risks to the outcome requires further validation, the merit of study concept, methodology and outcome measurements should be applicable in evolving regions and countries for evaluation of maternal-infant healthcare improvement taking SES into consideration.

## Conclusions

This cohort study provided a comprehensive and detailed data file of the outcome of livebirths and hospitalized neonates, depicting as NMR, IMR, and major causes of infant death associated with perinatal, neonatal and postneonatal risks in particular periods of infancy, in Huai’an, China. It reflects the progression and efficiency of contemporary maternal-infant healthcare with universal insurance policy in a particular regional livebirth population. The study concept, applicability and representativeness may be adopted and validated by other evolving regions or even amplified for provincial annual birth size (of 10-100 million residents in most of the 31 provinces/autonomous regions) for the same purpose.

## Supplementary Information

**Additional file 1.** Supplementary method for population attributable fraction (PAF) of polytomous variable

**Additional file 2 Table S1** Gestational age (GA) stratified rates of hospitalization and mortality

**Additional file 3 Table S2** Birthweight (BW) stratified rates of hospitalization and mortality

**Additional file 4 Table S3** Information of delivery room, in-hospital and out-of-hospital deaths

**Additional file 5 Table S4** Univariable analysis of perinatal risk factors for infant mortality at different periods by Cox regression model

## Data Availability

The datasets generated during and analyzed during the current study are not publicly available due to other concurrent studies based on the datasets but are available from the corresponding author on reasonable request.

## Abbreviations

AF: Amniotic fluid
aHR: Adjusted hazard ratio
BW: Birthweight
CI: Confidence interval
CNY: Chinese Yuan
DR: Delivery room
ELBW: Extremely low birthweight
EPT: Extreme preterm
GA: Gestational age
GDM: Gestational diabetes of mellitus
GDP: Gross domestic production
HR: Hazard ratio
ICD: International Statistical Classification of Diseases
HDP: Hypertensive disorder of pregnancy
IMR: Infant mortality rate
LBW: Low birthweight
MCHSS: Maternal and Child Health Surveillance System
NICU: Neonatal intensive care units
NMR: Neonatal mortality rate
PAF: Population attributable fraction
PND: Postnatal days
PROM: Prelabor rupture of membrane
RR: Relative risk
SES: Socioeconomic status
SGA: Small for gestational age
SUDI: Sudden unexpected death in infancy
VLBW: Very low birthweight
VPT: Very preterm
