# Supplementary material for: Perinatal Risks of Neonatal and Infant Mortalities in a Sub-provincial Region of China: A Livebirth Population-based Cohort Study
Source: BMC Pregnancy Childbirth. 2022 Apr 19;22:338. doi: 10.1186/s12884-022-04653-8 (PMC9020038; doi:10.1186/s12884-022-04653-8)
Supplement: Supplementary file 1 — Additional file 1. Supplementary method for population attributable fraction (PAF) of polytomous variable [file 12884_2022_4653_MOESM1_ESM.docx]

**Supplementary method for population attributable fraction (PAF) of polytomous variable.**

The equation was *PAF_i_*$=\frac{p_{i}({RR}_{i}-1)}{{1+\Sigma}_{i=1}^{i}p_{i}\left( {RR}_{i}-1 \right)}$ . RRi and pi respectively represented the relative risks and the prevalence rate in the target population for the i^th^ level of risk factors. Take the gestational age (GA) stratified infant mortality as example:

| **GA (week)** | **i** | **Infant livebirth, N** | **Infant death, n** | **Stratified death rate (‰)** | **p** | **RR (95% CI)** | **PAF**  **(%)** |
| --- | --- | --- | --- | --- | --- | --- | --- |
| 25-27 | 1 | 54 | 34 | 629.6 | 0.001 | 246.12 (167.54, 361.55) | 11.63 |
| 28-31 | 2 | 265 | 52 | 196.2 | 0.005 | 76.7 (55.15, 106.68) | 17.62 |
| 32-36 | 3 | 2195 | 37 | 16.9 | 0.037 | 6.59 (4.54, 9.56) | 10.78 |
| 37-38 | 4 | 12916 | 55 | 4.3 | 0.219 | 1.67 (1.20, 2.30) | 7.60 |
| 39-41 |  | 42998 | 110 | 2.6 |  | 1.00 (Reference) |  |
| > 42 | 5 | 628 | 3 | 4.8 | 0.011 | 1.87 (0.59, 5.88) | 0.48 |

PAF of GA 25-27 weeks:

PAF_1_ (%)=$\frac{p1*(RR1-1)}{1+ p1*(RR1-1) + p2*(RR2-1) + p3*(RR3-1) + p4*(RR4-1) + p5*(RR5-1)}$*100%=

$\frac{0.001*(246.12-1)}{1+ 0.001*(246.12-1) + 0.005*(76.7-1) + 0.037*(6.59-1) + 0.219*(1.67-1) +0.011*(1.87-1)}$*100%=11.63%

PAF of GA 28-31 weeks:

PAF_2_ (%)=$\frac{p2*(RR2-1)}{1+ p1*(RR1-1) + p2*(RR2-1) + p3*(RR3-1) + p4*(RR4-1) + p5*(RR5-1)}$*100%=

$\frac{0.005*(76.7-1)}{1+ 0.001*(246.12-1) + 0.005*(76.7-1) + 0.037*(6.59-1) + 0.219*(1.67-1) +0.011*(1.87-1)}$*100%=17.62%

PAF of GA 32-36 weeks:

PAF_3_ (%)=$\frac{p3*(RR3-1)}{1+ p1*(RR1-1) + p2*(RR2-1) + p3*(RR3-1) + p4*(RR4-1) + p5*(RR5-1)}$*100%=

$\frac{0.037*(6.59-1)}{1+ 0.001*(246.12-1) + 0.005*(76.7-1) + 0.037*(6.59-1) + 0.219*(1.67-1) +0.011*(1.87-1)}$*100%=10.78%

PAF of GA 37-38 weeks:

PAF_4_ (%)=$\frac{p1*(RR1-1)}{1+ p1*(RR1-1) + p2*(RR2-1) + p3*(RR3-1) + p4*(RR4-1) + p5*(RR5-1)}$*100%=

$\frac{0.219*(1.67-1)}{1+ 0.001*(246.12-1) + 0.005*(76.7-1) + 0.037*(6.59-1) + 0.219*(1.67-1) +0.011*(1.87-1)}$*100%=7.60%

PAF of GA > 42 weeks:

PAF_5_ (%)=$\frac{p1*(RR1-1)}{1+ p1*(RR1-1) + p2*(RR2-1) + p3*(RR3-1) + p4*(RR4-1) + p5*(RR5-1)}$*100%=

$\frac{0.011*(1.87-1)}{1+ 0.001*(246.12-1) + 0.005*(76.7-1) + 0.037*(6.59-1) + 0.219*(1.67-1) +0.011*(1.87-1)}$*100%=0.48%
