# Supplementary material for: Perinatal Risks of Neonatal and Infant Mortalities in a Sub-provincial Region of China: A Livebirth Population-based Cohort Study
Source: BMC Pregnancy Childbirth. 2022 Apr 19;22:338. doi: 10.1186/s12884-022-04653-8 (PMC9020038; doi:10.1186/s12884-022-04653-8)
Supplement: Supplementary file 2 — Additional file 2 Table S1 Gestational age (GA) stratified rates of hospitalization and mortality [file 12884_2022_4653_MOESM2_ESM.docx]

**Table S1** Gestational age (GA) stratified rates of hospitalization and mortality.

|  |  | **Hospitalization and mortality rates**  **with GA stratification (%)^a^** | | | |  | **Hospitalization and mortality rates**  **corrected by total livebirths (‰)^b^** | | | |
| --- | --- | --- | --- | --- | --- | --- | --- | --- | --- | --- |
| **GA (week)** | **Livebirths**  **(‰)^b^** | **Hospitalization** | **Deaths at**  **DR** | **Neonatal mortality** | **Infant mortality** |  | **Hospitalization** | **Deaths at**  **DR** | **Neonatal mortality** | **Infant mortality** |
| 25- | 2 (0.0) | 1 (50.0) | 1 (50.0) | 2 (100.0) | 2 (100.0) |  | 0.02 | 0.02 | 0.03 | 0.03 |
| 26- | 20 (0.3) | 11 (55.0) | 8 (40.0) | 14 (70.0) | 15 (75.0) |  | 0.19 | 0.14 | 0.24 | 0.25 |
| 27- | 32 (0.5) | 28 (87.5) | 3 (9.4) | 12 (37.5) | 17 (53.1) |  | 0.47 | 0.05 | 0.20 | 0.29 |
| 28- | 55 (0.9) | 43 (78.2) | 7 (12.7) | 20 (36.4) | 21 (38.2) |  | 0.73 | 0.12 | 0.34 | 0.36 |
| 29- | 51 (0.9) | 51 (100.0) | 0 | 13 (25.5) | 13 (25.5) |  | 0.86 | 0 | 0.22 | 0.22 |
| 30- | 58 (1.0) | 54 (93.1) | 1 (1.7) | 10 (17.2) | 11 (19.0) |  | 0.91 | 0.02 | 0.17 | 0.19 |
| 31- | 101 (1.7) | 93 (92.1) | 3 (3.0) | 5 (5.0) | 7 (6.9) |  | 1.57 | 0.05 | 0.08 | 0.12 |
| 32- | 163 (2.8) | 142 (87.1) | 2 (1.2) | 9 (5.5) | 9 (5.5) |  | 2.40 | 0.03 | 0.15 | 0.15 |
| 33- | 245 (4.1) | 217 (88.6) | 0 | 6 (2.4) | 7 (2.9) |  | 3.67 | 0 | 0.10 | 0.12 |
| 34- | 331 (5.6) | 310 (93.7) | 1 (0.3) | 6 (1.8) | 6 (1.8) |  | 5.25 | 0.02 | 0.10 | 0.10 |
| 35- | 478 (8.1) | 464 (97.1) | 0 | 3 (0.6) | 4 (0.8) |  | 7.86 | 0 | 0.05 | 0.07 |
| 36- | 978 (16.6) | 527 (53.9) | 1 (0.1) | 9 (0.9) | 11 (1.1) |  | 8.92 | 0.02 | 0.15 | 0.19 |
| 37- | 3199 (54.2) | 671 (21.0) | 1 (0.0) | 18 (0.6) | 22 (0.7) |  | 11.36 | 0.02 | 0.30 | 0.37 |
| 38- | 9717 (164.5) | 1229 (12.6) | 3 (0.0) | 23 (0.2) | 33 (0.3) |  | 20.81 | 0.05 | 0.39 | 0.56 |
| 39- | 19775 (334.9) | 1989 (10.1) | 2 (0.0) | 27 (0.1) | 52 (0.3) |  | 33.68 | 0.03 | 0.46 | 0.88 |
| 40- | 17192 (291.1) | 1592 (9.3) | 2 (0.0) | 28 (0.2) | 50 (0.3) |  | 26.96 | 0.03 | 0.47 | 0.85 |
| 41- | 6031 (102.1) | 495 (8.2) | 0 | 6 (0.1) | 8 (0.1) |  | 8.38 | 0 | 0.10 | 0.14 |
| 42- | 578 (9.8) | 41 (7.1) | 0 | 2 (0.3) | 3 (0.5) |  | 0.69 | 0 | 0.03 | 0.05 |
| 43- | 50 (0.9) | 2 (4.0) | 0 | 0 | 0 |  | 0.03 | 0 | 0 | 0 |
| Total | 59056 | 7960 (13.5) | 35 (0.1) | 213 (0.4) | 291 (0.5) |  | 134.79 | 0.59 | 3.61 | 4.93 |

Abbreviations: GA, gestational age; DR, delivery room.

Values are n or n (ratio in % or ‰).

a. Ratio refers to percentage (%) of subtotal livebirths in each GA stratum.

b. Ratio refers to per thousand (‰) of total livebirths (59056).
