# Supplementary material for: Perinatal Risks of Neonatal and Infant Mortalities in a Sub-provincial Region of China: A Livebirth Population-based Cohort Study
Source: BMC Pregnancy Childbirth. 2022 Apr 19;22:338. doi: 10.1186/s12884-022-04653-8 (PMC9020038; doi:10.1186/s12884-022-04653-8)
Supplement: Supplementary file 3 — Additional file 3 Table S2 Birthweight (BW) stratified rates of hospitalization and mortality [file 12884_2022_4653_MOESM3_ESM.docx]

**Table S2** Birthweight (BW) stratified rates of hospitalization and mortality.

|  |  | **Hospitalization and mortality rates**  **with BW stratification (%)^a^** | | | |  | **Hospitalization and mortality rates**  **corrected by total livebirths (‰)^b^** | | | |
| --- | --- | --- | --- | --- | --- | --- | --- | --- | --- | --- |
| **BW (g)** | **Livebirths**  **(‰)^b^** | **Hospitalization** | **Deaths at**  **DR** | **Neonatal mortality** | **Infant mortality** |  | **Hospitalization** | **Deaths at**  **DR** | **Neonatal mortality** | **Infant mortality** |
| 500- | 12 (0.2) | 1 (8.3) | 11 (91.7) | 12 (100.0) | 12 (100.0) |  | 0.02 | 0.19 | 0.20 | 0.20 |
| 750- | 22 (0.4) | 19 (86.4) | 3 (13.6) | 13 (59.1) | 16 (72.7) |  | 0.32 | 0.05 | 0.22 | 0.27 |
| 1000- | 84 (1.4) | 70 (83.3) | 4 (4.8) | 22 (26.2) | 27 (32.1) |  | 1.19 | 0.07 | 0.37 | 0.46 |
| 1250- | 128 (2.2) | 113 (88.3) | 1 (0.8) | 22 (17.2) | 23 (18.0) |  | 1.91 | 0.02 | 0.37 | 0.39 |
| 1500- | 172 (2.9) | 153 (89.0) | 1 (0.6) | 12 (7.0) | 12 (7.0) |  | 2.59 | 0.02 | 0.20 | 0.20 |
| 1750- | 276 (4.7) | 237 (85.9) | 1 (0.4) | 13 (4.7) | 13 (4.7) |  | 4.01 | 0.02 | 0.22 | 0.22 |
| 2000- | 506 (8.6) | 373 (73.7) | 3 (0.6) | 11 (2.2) | 15 (3.0) |  | 6.32 | 0.05 | 0.19 | 0.25 |
| 2250- | 648 (11.0) | 392 (60.5) | 1 (0.2) | 10 (1.5) | 11 (1.7) |  | 6.64 | 0.02 | 0.17 | 0.19 |
| 2500- | 1970 (33.4) | 603 (30.6) | 4 (0.2) | 21 (1.1) | 26 (1.3) |  | 10.21 | 0.07 | 0.36 | 0.44 |
| 2750- | 3584 (60.7) | 655 (18.3) | 1 (0.0) | 13 (0.4) | 15 (0.4) |  | 11.09 | 0.02 | 0.22 | 0.25 |
| 3000- | 11057 (187.2) | 1317 (11.9) | 2 (0.0) | 11 (0.1) | 31 (0.3) |  | 22.30 | 0.03 | 0.19 | 0.52 |
| 3250- | 10771 (182.4) | 1123 (10.4) | 0 | 17 (0.2) | 25 (0.2) |  | 19.02 | 0 | 0.29 | 0.42 |
| 3500- | 14364 (243.2) | 1447 (10.1) | 3 (0.0) | 16 (0.1) | 32 (0.2) |  | 24.50 | 0.05 | 0.27 | 0.54 |
| 3750- | 7360 (124.6) | 704 (9.6) | 0 | 14 (0.2) | 20 (0.3) |  | 11.92 | 0 | 0.24 | 0.34 |
| 4000- | 5453 (92.3) | 470 (8.6) | 0 | 2 (0.0) | 8 (0.1) |  | 7.96 | 0 | 0.03 | 0.14 |
| 4250- | 1505 (25.5) | 136 (9.0) | 0 | 3 (0.2) | 3 (0.2) |  | 2.30 | 0 | 0.05 | 0.05 |
| 4500- | 750 (12.7) | 88 (11.7) | 0 | 1 (0.1) | 2 (0.3) |  | 1.49 | 0 | 0.02 | 0.03 |
| 4750- | 194 (3.3) | 24 (12.4) | 0 | 0 | 0 |  | 0.41 | 0 | 0 | 0 |
| >5000 | 200 (3.4) | 35 (17.5) | 0 | 0 | 0 |  | 0.59 | 0 | 0 | 0 |
| Total | 59 056 | 7960 (13.5) | 35 (0.1) | 213 (0.4) | 291 (0.5) |  | 134.79 | 0.59 | 3.61 | 4.93 |

Abbreviations: BW, birthweight; DR, delivery room.

Values are n or n (ratio in % or ‰).

a. Ratio refers to percentage (%) of subtotal livebirths in each BW stratum.

b. Ratio refers to per thousand (‰) of total livebirths (59056).
