# Supplementary material for: Perinatal Risks of Neonatal and Infant Mortalities in a Sub-provincial Region of China: A Livebirth Population-based Cohort Study
Source: BMC Pregnancy Childbirth. 2022 Apr 19;22:338. doi: 10.1186/s12884-022-04653-8 (PMC9020038; doi:10.1186/s12884-022-04653-8)
Supplement: Supplementary file 4 — Additional file 4 Table S3 Information of delivery room, in-hospital and out-of-hospital deaths [file 12884_2022_4653_MOESM4_ESM.docx]

**Table S3** Information of delivery room, in-hospital and out-of-hospital deaths.

|  | **Deaths at**  **DR** | **In-hospital deaths** | **Out-of-hospital deaths** | | **Total deaths** | |  |
| --- | --- | --- | --- | --- | --- | --- | --- |
| N | 35 | 168 | 88 | | 291 | |  |
| GA 25-27 (week) | 12 (34.3) | 22 (13.1) | 0 | | 34 (11.7) | |  |
| 28-31 | 11 (31.4) | 39 (23.2) | 2 (2.3) | | 52 (17.9) | |  |
| 32-36 | 4 (11.4) | 28 (16.7) | 5 (5.7) | | 37 (12.7) | |  |
| 37-38 | 4 (11.4) | 27 (16.1) | 24 (27.3) | | 55 (18.9) | |  |
| 39-41 | 4 (11.4) | 50 (29.8) | 56 (63.6) | | 110 (37.8) | |  |
| >42 | 0 | 2 (1.2) | 1 (1.1) | | 3 (1.0) | |  |
| BW<1000 (g) | 14 (40.0) | 14 (8.3) | 0 | | 28 (9.6) | |  |
| 1000-1499 | 5 (14.3) | 44 (26.2) | 1 (1.1) | | 50 (17.2) | |  |
| 1500-2499 | 6 (17.1) | 34 (20.2) | 11 (12.5) | | 51 (17.5) | |  |
| 2500-3999 | 10 (28.6) | 71 (42.3) | 68 (77.3) | | 149 (51.2) | |  |
| >4000 | 0 | 5 (3.0) | 8 (9.1) | | 13 (4.5) | |  |
| PND 0-6 (days) | 35 (100.0) | 108 (64.3) | 10 (11.4) | | 153 (52.6) | |  |
| 7-27 | 0 | 50 (29.8) | 10 (11.4) | | 60 (20.6) | |  |
| 28-364 | 0 | 10 (6.0) | 68 (77.3) | | 78 (26.8) | |  |
| Cause of infant deaths | |  | |  | |  | |
| Perinatal conditions | 28 (80.0) | 116 (69.0) | 11 (12.5) | | 155 (53.3) | |  |
| Congenital anomalies | 7 (20.0) | 52 (31.0) | 31 (35.2) | | 90 (30.9) | |  |
| SUDI | 0 | 0 | 35 (39.8) | | 35 (12.0) | |  |
| Other causes | 0 | 0 | 11 (12.5) | | 11 (3.8) | |  |

Abbreviations: DR, delivery room; GA, gestational age; BW, birthweight; PND, postnatal days; SUDI, sudden unexpected death in infancy.

Values are given in n (%), refer to all deaths in respective columns.
