# Supplementary material for: Perinatal Risks of Neonatal and Infant Mortalities in a Sub-provincial Region of China: A Livebirth Population-based Cohort Study
Source: BMC Pregnancy Childbirth. 2022 Apr 19;22:338. doi: 10.1186/s12884-022-04653-8 (PMC9020038; doi:10.1186/s12884-022-04653-8)
Supplement: Supplementary file 5 — Additional file 5 Table S4 Univariable analysis of perinatal risk factors for infant mortality at different periods by Cox regression model [file 12884_2022_4653_MOESM5_ESM.docx]

**Table S4** Univariable analysis of perinatal risk factors for infant mortality at different periods by Cox regression model.

|  | **Crude HR (95% CI)** | | |  |
| --- | --- | --- | --- | --- |
|  | **Early neonatal death** | **Late neonatal death** | **Postneonatal death** | |
| Maternal age < 20 (y) | 1.87 (0.95, 3.67) | 2.36 (0.85, 6.57) | 0.39 (0.05, 2.78) | |
| 20-34 | 1.00 (Reference) | 1.00 (Reference) | 1.00 (Reference) | |
| > 35 | 1.29 (0.77, 2.16) | 2.52 (1.30, 4.87) | 1.20 (0.58, 2.48) | |
| Rural residency | 1.82 (1.28, 2.59) | 1.43 (0.83, 2.44) | 1.51 (0.94, 2.44) | |
| Education > 9 y | 0.71 (0.51, 1.00) | 0.72 (0.42, 1.25) | 0.65 (0.40, 1.06) | |
| Inadequate prenatal care | 1.14 (0.83, 1.57) | 0.90 (0.54, 1.51) | 1.16 (0.74, 1.81) | |
| Multipara | 1.34 (0.96, 1.88) | 1.25 (0.73, 2.12) | 1.08 (0.68, 1.70) | |
| HDP | 2.92 (1.83, 4.68) | 2.58 (1.17, 2.57) | 1.93 (0.89, 4.19) | |
| GDM | 2.63 (1.16, 5.96) | 1.10 (0.15, 7.92) | 1.70 (0.42, 6.93) | |
| Anemia | 2.29 (1.48, 3.54) | 1.37 (0.59, 3.18) | 1.21 (0.56, 2.64) | |
| PROM | 3.06 (2.10, 4.46) | 4.80 (2.79, 8.27) | 1.19 (0.57, 2.47) | |
| Placenta | 4.98 (2.70, 9.19) | 4.63 (1.68, 12.76) | 2.60 (0.82, 8.24) | |
| Umbilical cord | 3.68 (2.25, 6.02) | 4.27 (2.03, 8.99) | 0.73 (0.18, 2.98) | |
| Antenatal steroids | 15.66 (10.78, 22.74) | 15.83 (8.70, 28.80) | 6.01 (2.89, 12.48) | |
| Fetal distress | 4.10 (1.92, 8.75) | 1.46 (0.20, 10.50) | 1.11 (0.16, 8.01) | |
| Cesarean delivery | 0.67 (0.49, 0.93) | 0.84 (0.51, 1.40) | 1.15 (0.73, 1.80) | |
| AF contamination | 2.15 (1.49, 3.12) | 2.06 (1.13, 3.74) | 1.23 (0.67, 2.28) | |
| Multiple births | 7.36 (4.64, 11.66) | 6.16 (2.80, 13.56) | 7.73 (4.08, 14.62) | |
| Male | 1.09 (0.79, 1.50) | 0.87 (0.52, 1.44) | 1.47 (0.93, 2.33） | |
| GA 25-27 (week) | 538.49  (321.89, 900.86) | 488.11  (193.73, 1229.83) | 251.63  (107.77, 587.50) | |
| 28-31 | 142.11 (90.95, 222.04) | 138.76 (67.99, 283.21) | 16.38 (5.91, 45.39) | |
| 32-36 | 11.44 (6.99, 18.73) | 8.81 (3.83, 20.26) | 1.62 (0.59, 4.49) | |
| 37-38 | 2.17 (1.35, 3.49) | 2.41 (1.18, 4.91) | 0.95 (0.53, 1.73) | |
| 39-41 | 1.00 (Reference) | 1.00 (Reference) | 1.00 (Reference) | |
| > 42 | - | 7.61 (1.77, 32.79) | 1.40 (0.19, 10.14) | |
| BW < 1000 (g) | 749.93  (458.34, 1227.02) | 738.19  (256.05, 2128.21) | 391.02  (122.38, 1249.34) | |
| 1000-1499 | 116.78 (76.71, 177.78) | 140.80 (70.41, 181.55) | 31.69 (13.67, 73.50) | |
| 1500-2499 | 13.64 (8.88, 20.96) | 20.90 (11.10, 39.33) | 2.77 (1.11, 2.90) | |
| 2500-3999 | 1.00 (Reference) | 1.00 (Reference) | 1.00 (Reference) | |
| > 4000 | 0.19 (0.04, 0.73) | 1.01 (0.35, 2.91) | 0.74 (0.34, 1.63) | |
| SGA | 2.81 (1.70, 4.65) | 2.05 (0.82, 5.12) | 1.55 (0.62, 3.82) | |
| Apgar 5-min < 7 | 173.44 (126.24, 238.29) | 23.22 (29.26, 96.80) | 17.76 (8.17, 38.60) | |
| DR resuscitation | 15.21 (10.87, 21.29) | 10.33 (5.76, 18.54) | 4.07 (2.03, 8.15) | |
| Congenital anomalies | 32.56 (23.05, 46.00) | 97.52 (58.64, 162.17) | 19.66 (11.20, 34.52) | |
| Hospitalization | 16.50 (11.60, 23.47) | 91.36 (33.13, 251.94) | 5.63 (3.61, 8.78) | |

Abbreviations: HR, hazard ratio, in comparison with the subgroups in reference; CI, confidence interval; HDP, hypertensive disorder of pregnancy; GDM, gestational diabetes of mellitus; PROM, prelabor rupture of membrane; AF, amniotic fluid; GA, gestational age; BW, birthweight; SGA, small for gestational age; DR, delivery room.
